# Proliferation and Apoptosis of Cat (*Felis catus*) Male Germ Cells during Breeding and Non-Breeding Seasons

**DOI:** 10.3390/vetsci9080447

**Published:** 2022-08-20

**Authors:** Luisa Valentini, Rosa Zupa, Chrysovalentinos Pousis, Rezart Cuko, Aldo Corriero

**Affiliations:** Department of Emergency and Organ Transplantation, Section of Veterinary Clinics and Animal Production, University of Bari Aldo Moro, 70010 Bari, Italy

**Keywords:** feline, testicle, orchiectomy, spermatogenesis, seasonal breeding

## Abstract

**Simple Summary:**

Spermatogenesis is a complex process through which male gametes, spermatozoa, are produced starting from stem germ cells called spermatogonia. The existing information on cat spermatogenesis is limited and somewhat contradictory. In fact, although this species is considered a long-day breeder with a reproduction period starting when the day length increases and ending in late autumn, spermatogenesis and sperm production occur throughout the year. In order to assess whether cat spermatogenesis is modulated according to a season pattern, we analyzed testes taken from feral cats orchiectomized during reproductive (February–July) and non-reproductive (November and December) periods. The results of the analyses carried out in the present study showed that spermatogonial proliferation was more intense during the reproductive period and germ cell death via apoptosis (a programmed form of cell death) increased during the non-reproductive period. Our results confirm the hypothesis that cat spermatogenesis is seasonally modulated through changes of germ cell proliferation and apoptosis, according to a common paradigm of seasonally breeding species.

**Abstract:**

The domestic cat (*Felis catus*) is a seasonal-breeding species whose reproductive period starts when the day length increases. Since the existing information on cat spermatogenesis is limited and somewhat contradictory, in the present study, germ cell proliferation and apoptosis in feral adult tomcats orchiectomized during reproductive (reproductive group, RG; February–July) and non-reproductive (non-reproductive group, NRG; November and December) seasons were compared. Cross-sections taken from the middle third of the left testis were chemically fixed and embedded in paraffin wax. Histological sections were processed for the immunohistochemical detection of proliferating germ cells (PCNA) and for the identification of apoptotic cells (TUNEL method). The percentage of PCNA-positive spermatogonia was higher in the RG than in the NRG. On the contrary, germ cell apoptosis was higher in the NRG than in the RG. Our results confirm that cat spermatogenesis is modulated on a seasonal basis and suggests that spermatogenesis control involves changes in germ cell proliferation and apoptosis according to a common paradigm of seasonally breeding species.

## 1. Introduction

Spermatogenesis is a complex process occurring in testes through which spermatozoa are produced starting from stem germ cells called spermatogonia [1]. In mammals, spermatogenesis occurs in seminiferous tubules and is a cyclical process resulting in a massive and continuous sperm production [2]. An ordered repetitive sequence of specific cellular associations is commonly observed along the seminiferous tubules [2,3,4], and each cellular association constitutes a stage or phase of the spermatogenesis. Any specific area of the seminiferous epithelium undergoes a spermatogenic cycle, i.e., a sequence of events that starts with the appearance of a specific stage of the spermatogenesis and leads to the reappearance of the same stage.

Although the domestic cat (*Felis catus*) is one of the most popular pets, the existing knowledge of its spermatogenesis is limited [4]. It is widely known that the cat is a long-day breeder species whose reproductive activity is not continuous because the female (queen) is a seasonally polyestrous and an induced ovulator [5], and, in temperate climate zones of the Northern Hemisphere, breeding season begins during February/March and continues until September [6]. However, due to the presence of contradictory literature data, cat spermatogenesis needs further investigation. It has been reported that sperm production in cats does not show seasonal variations [4,7,8]. The available histological studies on cat spermatogenesis have been based on the assumption that spermatozoa are produced throughout the year, so that no seasonal differences in the activity of the germinal epithelium occur [4,9]. On the contrary, other studies have reported seasonal changes in testosterone concentrations [10,11], testis mass [10,12], germ cell tubule composition (spermatogenic stages) and sperm quality [11,12]. Moreover, in testes obtained from free-roaming tomcats living in the region of Berlin (Germany), significant variations were observed in the spermatogenic activity and in the percentage of motile sperm between spring and autumn months [12]. 

In mammals [13,14,15] and in non-mammalian vertebrates with seasonal reproductive cycles, including fish [16,17,18], reptiles [19,20], amphibians [21,22] and birds [23], the spermatogenesis process follows a seasonal pattern of germ cell proliferation and apoptosis, i.e., the two mechanisms responsible for regulating sperm output, keeping the number of stem germ cell stable to avoid testis depletion and assuring the correct Sertoli cells/germ cells ratio [14,15,24,25]. 

The aim of the present study was to describe the changes in germ cell proliferation and apoptosis occurring in the testis of stray tomcats living in urban colonies and orchiectomized during reproductive and non-reproductive seasons in order to improve our understanding of the spermatogenesis process in this species.

## 2. Material and Methods

### 2.1. Sample Collection

The study was authorized by the Ethics Committee for Veterinary Clinical and Zootechnical Studies of the Department of Emergency and Organ Transplantation of the University of Bari Aldo Moro (authorization # 04/2021). 

For the present study, testes from 16 cats that underwent surgical orchiectomy were used. Testes were divided in two groups, according to the season during which cats were orchiectomized: reproductive group (RG; n = 8), including samples taken between March and July; and non-reproductive group (NRG; n = 8), including samples taken between November and December. 

The testis samples were provided from the Veterinary Service (Servizio Igiene e Assistenza Veterinaria Area A—Sanità Animale, S.I.A.V. “A”) of the Local Health Authority Veterinary Service (ASL BA) in charge of stray cat reproduction control. No tomcats were specifically sterilized for the present research. 

All the testes were taken from adult feral cats belonging to colonies settled in the urban area of Bari, Italy. Only testes belonging to tomcats with evident sexual secondary characters (well-developed penis spines, stout necks and stud jowls) and that did not show evident pathologies during the routine preoperative clinic visit were recruited for the study. Moreover, all testes that showed anatomical–histopathological lesions were excluded from the present study. 

### 2.2. Testis Histology, Immunohistochemical Detection of Proliferating Germ Cells and Apoptosis

Cross-sections (0.5 cm tick) were taken from the middle third of the left testis of each cat, fixed in Bouin’s solution for 4 h, dehydrated in increasing ethanol concentrations, clarified in xylene and embedded in paraffin wax. Sections (thickness: four μm) were cut by using a Leitz 1510 (Ernst Leitz Wetzlar, Germany) microtome and processed for the immunohistochemical detection of proliferating germ cells and for the identification of apoptotic cells. For the identification of proliferating germ cells, sections were deparaffinized and re-hydrated, and endogenous peroxidase was inhibited by treatment for 10 min with 3% H_2_O_2_, followed by rinsing with distilled water and phosphate-buffered saline (PBS, 0.01 M, pH 7.4, containing 0.15 M NaCl). Sections were then incubated for 30 min at room temperature in normal horse serum (NHS; Vector, Burlingame, CA, USA) to block non-specific binding sites for immunoglobulins and then incubated overnight in a moist chamber at 4 °C, with monoclonal antibodies raised against proliferating cell nuclear antigen (PCNA) (Santa Cruz Biotechnology Inc., Dallas, TX, USA), a polymerase delta accessory protein that is synthesized in late G1 and S phases of the cell cycle and proved to be a reliable nuclear marker of cell proliferation. Anti-PCNA antibodies were diluted 1:50 in PBS containing 0.1% bovine serum albumin (BSA) (Sigma-Aldrich, Milan, Italy). After rinsing for 10 min in PBS, immunohistochemical visualization was obtained by using the Vectastain Universal Elite Kit (Vector, Burlingame, CA, USA). This method uses the avidin–biotin–peroxidase complex (ABC) procedure. Peroxidase activity was then visualized by incubating for 10 min with a Vector 3,3’-diaminobenzidine (DAB) Peroxidase Substrate Kit (Vector, Burlingame, CA, USA), which produces a brown precipitate. Finally, to obtain a weak nuclear background staining, sections were immersed for 30 s in hematoxylin. To confirm the specificity of the immunoreaction, routine control-staining procedures were carried out by replacement of the primary antibody with NHS or PBS.

The identification of apoptotic germ cells was performed by using the terminal deoxynucleotidyl transferase–mediated d’UTP nick end labeling (TUNEL) method with an in situ Cell Death Detection Kit, AP (Roche Diagnostics, Mannheim, Germany) that was used according to the manufacturer’s instructions. Sections were re-hydrated through graded ethanol solutions and then incubated in a permeabilization solution of 0.1% Triton X-100 in 0.1% sodium citrate for 8 min, at 37 °C, prior to incubation with the reaction mixture. In order to minimize the background staining, the terminal deoxynucleotidyl transferase was diluted 1:2 in TUNEL Dilution Buffer (Roche Diagnostics, Mannheim, Germany). A ready-to-use solution of nitro-blue tetrazolium chloride/5-bromo-4-chloro-3’-indolyphosphate p-toluidine salt (NBT/BCIP) (Roche Diagnostics, Mannheim, Germany) was used as a substrate for the signal conversion. Finally, in order to obtain a weak nuclear background staining, sections were treated for 30 s with Fast Red (Vector, Burlingame, CA, USA).

### 2.3. Quantification of Germ Cell Proliferation and Apoptosis

For each specimen, PCNA-positive and PCNA-negative spermatogonia were counted in at least 50 seminiferous tubules per specimen showing a round profile on microphotographs taken with a digital camera (K3, Leica, Wetzlar, Germany) connected to a light microscope (DMRB, Leica, Wetzlar, Germany), using image-analysis software (LAS X, Wetzlar, Germany). The rate of spermatogonial proliferation was expressed as the percentage of PCNA-positive spermatogonia on the total number of counted spermatogonia.

Apoptotic cells and apoptotic bodies were counted in at least 50 cross-sectioned tubules showing a round profile per specimen. The mean number of apoptotic structures per seminiferous tubule was calculated as total number of apoptotic cells/bodies on total number of analyzed seminiferous tubules, and the percentage of seminiferous tubules containing at least one apoptotic cell or apoptotic body was also recorded. An apoptotic index was calculated as the mean number of apoptotic structures per seminiferous tubule multiplied by the frequency of seminiferous tubules containing at least one apoptotic cell. 

### 2.4. Statistical Analysis

The percentage of PCNA-positive spermatogonia and the percentage of seminiferous tubules containing at least one apoptotic structure were compared by chi-square test in RG versus NRG. The mean number of apoptotic structures did not show normal distributions (assessed through the Shapiro–Wilk W test); hence, the differences in the number of apoptotic structures and apoptotic index were assessed by using the Mann–Whitney U test. Statistical analyses were performed by using MS Office Excel 365, and statistical significance was accepted for *p* < 0.05. Results were expressed as mean ± SD.

## 3. Results

**Figure 1:**
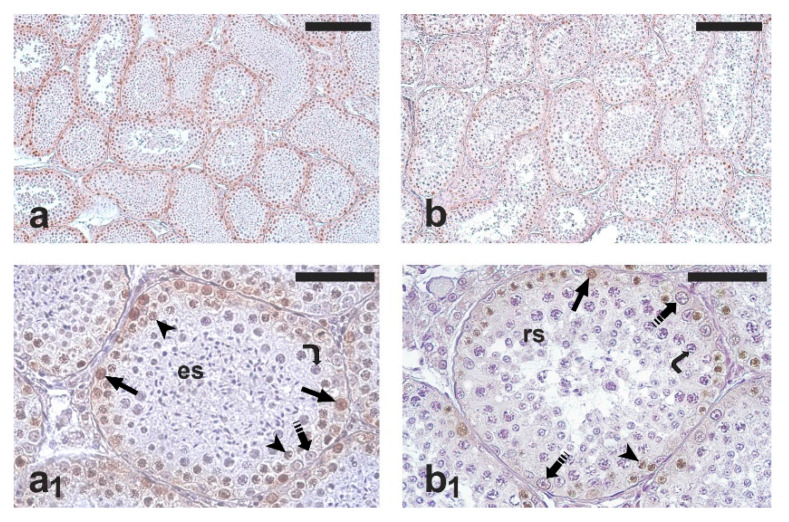
Micrographs of testis sections from cats orchiectomized during the reproductive (**left**) and non-reproductive period (**right**) that were immunostained with antibodies against proliferating cell nuclear antigen (PCNA). Dividing germ cells, stained in brown, are localized in the peripheral region of seminiferous tubules. (**a**,**b**) Low-magnification micrographs showing the general pattern of anti-PCNA immunostaining. (**a_1_**) Cross-section of a seminiferous tubule containing spermatogonia, spermatocytes, and elongated spermatids—a germ cell composition corresponding to the middle stage group (spermatogenic stages VI–VIII) of Xu et al. (2021) [2]. (**b_1_**) Cross-section of a seminiferous tubule containing spermatogonia, spermatocytes, and rounded spermatids—a germ cell composition corresponding to the early stage group (spermatogenic stages I–V) of Xu et al. (2021) [2]. A higher number of PCNA-positive cells is visible in (**a_1_**) and in (**b_1_**). Arrow, PCNA-positive spermatogonium; dashed arrow, PCNA-negative spermatogonium; arrowhead, PCNA-positive spermatocyte; curved arrow, PCNA-negative spermatocyte; es, elongated spermatids; rs, round spermatids. Scale bars = 200 μm in (**a**,**b**) and 50 μm in (**a_1_**,**b_1_**).

As expected, all the testis samples examined showed active spermatogenesis (Figure 1a,b). PCNA-positive cells were observed in all the seminiferous tubules; occasionally, PCNA-positive cells were found also in the intertubular compartment. The immunohistochemical staining mainly involved spermatogonia; weakly stained primary spermatocytes were also observed (Figure 1a_1_,b_1_). However, only spermatogonia were counted for the comparative analysis of germ cell proliferation in the two groups. The percentage of PCNA-positive spermatogonia was significantly higher in the RG than in the NRG (77.8 ± 6.7 vs. 64.0 ± 6.3; *p* < 0.05).

**Figure 2:**
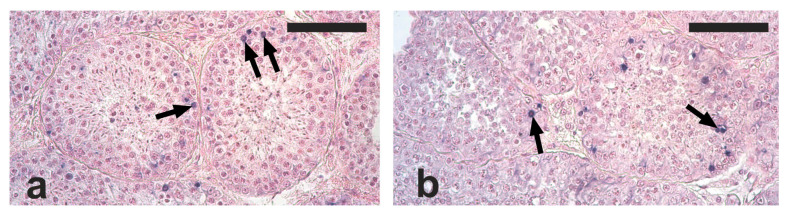
Micrographs of testis sections from cats orchiectomized during the reproductive (**a**) and non-reproductive (**b**) seasons stained with the terminal deoxynucleotidyl transferase-mediated d’UTP nick end labeling (TUNEL) method and counterstained with Fast Red. Apoptotic cells and apoptotic bodies (arrows) are visible within the tubular compartment as blue spots. Scale bars = 100 μm.

Apoptotic cells and apoptotic bodies were found in the tubular compartment of all the analyzed testis sections. The distribution of apoptotic structures was not homogenous, since tubules with no, one, or several apoptotic structures were observed. Based on their localization within tubule sections, the observed apoptotic structures were mostly represented by died spermatogonia and spermatocytes (Figure 2a,b).

The mean number of apoptotic structures was lower, although not significantly, in the RG compared with the NRG (2.2 ± 1.5 vs. 3.0 ± 0.9; *p* = 0.19). The percentage of seminiferous tubules containing at least one apoptotic structure was significantly lower in the RG than in the NRG (57.9 ± 23.0 vs. 75.1 ± 12.3; *p* < 0.05). Among TUNEL-positive seminiferous tubules, 20% showed only one apoptotic structure, and the remaining had two or more apoptotic structures. The apoptotic index was lower, although not significantly, in the RG than in the NRG (1.5 ± 1.3 vs. 2.4 ± 1.0; *p* = 0.17).

## 4. Discussion

The knowledge of feline spermatogenesis may provide elements that contribute to improve our understanding of the general paradigm of mammalian reproduction and may have applied implications in biomedical sciences and veterinary practice [4]. However, most of the available data on feline spermatogenesis originated from studies based on owned tomcats, probably held under artificial illumination. Moreover, some of the available studies apparently overlooked seasonality of cats’ reproductive biology, although it is widely known that tomcat reproductive activity is strongly affected by photoperiod, social context, and estrous queen contiguity.

In the present study, we selected, over a wider available collection, only testes taken from feral cats living in urban colonies that were recruited into a population-control program of the Apulian Regional Health Service and did not show evident pathologies during the routine preoperative clinic visit or anatomical/histological alterations. Moreover, testes in active spermatogenesis belonging to animals with clearly developed secondary sexual characters were selected. This rigorous selection much reduced the number of the available samples; however, it assured that the outputs of the study were not biased by conditions that could have altered the natural reproductive activity, and then represented genuine elements of cat natural spermatogenesis.

We showed that significant seasonal changes occur in spermatogonial proliferation of adult feral cats. In fact, a much higher percentage of dividing spermatogonia was observed in testis samples taken from tomcats orchiectomized during reproductive months. A wide body of the literature data clearly indicate that, both in mammals and non-mammalian vertebrates with marked seasonality in mating and reproduction, the spermatogenic activity shows seasonal changes [13,14,15,16,17,18,19,20,21,22,23]. The testicular functions in vertebrates are controlled by the hypothalamic–pituitary–gonadal (HPG) axis. Environmental and social factors, as well as the individual’s energy state, drive the hypothalamic release of the gonadotropin-releasing hormone (GnRH), which stimulates gonadotropin secretion by the pituitary. Gonadotropins, in turn, stimulate sex steroids synthesis and secretion [26,27]. In mammals, spermatogonial proliferation and progress toward meiosis are both stimulated by sex steroids, particularly testosterone [14]. Blottner and Jewgenow (2007) [12] and Kirkpatrick (2011) [10] documented seasonal changes in testicular testosterone concentrations in feral cats, with higher concentrations in spring months and reduced concentrations in autumn, associated with a reduction of testis mass. Moreover, Blottner and Jewgenow (2007) [12] also reported significant changes in the primary spermatocytes-to-spermatids ratio between the two periods, and Stornelli et al. (2009) [28] found seasonal changes in the proportion of seminiferous tubules at different stages of spermatogenesis. Taken together, the results of the present study and the literature data suggest that the modulation of spermatogonial proliferation might represent, also in mammal species whose spermatogenesis and sperm production are extended throughout the year, a conserved mechanism for limiting the energetic investment when reproductive success is unlikely.

Apoptosis, an integral component of normal testicular function in mammals, plays an important role in limiting germ cell population, assuring the optimal Sertoli-cell-to-germ-cell ratio, and preventing maturation of aberrant germ cells [14,29]. In seasonally breeding mammals, during the non-reproductive period, testes undergo a variable degree of mass reduction due to both cell size reduction and cell death [14,30]. In general, apoptotic cell death intensifies during testicular regression and reduces throughout testicular recrudescence and breeding season [14,15].

In the cat, male germ apoptosis has been documented at specific stages of spermatogenesis, concomitantly with DNA synthesis. Interestingly, cat testicular apoptosis occurs at the same stage as those reported for rabbit and rodents [9]. In cats living in Poland that underwent surgical castration (the origin of the cats was not specified, but they likely were client-owned domestic animals), Siemieniuch (2008) [31] reported a higher percentage of individuals showing intense testicular apoptosis in autumn/winter (15.2%) than in spring/summer (10.1%). An augmented expression of apoptosis-related genes was reported in testis samples from owned cats orchiectomized during the non-reproductive season in Poland [32]. In the present study, we found sparse apoptotic germ cells, apparently spermatogonia and spermatocytes, in all the examined testes. The percentage of seminiferous tubule sections containing apoptotic cells showed statistically significant variations between the reproductive and non-reproductive group, whereas the other two parameters, i.e., the mean number of apoptotic cells per tubule section and the apoptotic index, failed to reach statistically significant changes, possibly due to the abovementioned limitation in sample numerosity. Nevertheless, the results of the present study suggest that germ cell apoptosis is one of the factors involved in the seasonal modulation of spermatogenesis in cat. Gonadotropins and testosterone are known germ cell survival factors in mammals, and the withdrawal of gonadotropins and testosterone has been shown to induce testicular apoptosis [14]; thus, it can be supposed that, similar to mammal species with marked seasonal reproductive cycles, cat spermatogenic activity is modulated through changes in testosterone synthesis and secretion, which, in turn, regulate germ cell proliferation and apoptosis rates.

In conclusion, the present study confirms that cat spermatogenesis is modulated on a seasonal basis and suggests that spermatogenesis control may be exerted by the HPG axis through changes in germ cell proliferation and apoptosis, according to a common paradigm of vertebrate species with restricted breeding season. In the tomcat, the social context, and particularly the presence of reproductively active queen contiguity, may also play an important role, through visual and pheromonal signaling, in the modulation of spermatogenesis. The results of this study suggest that purebred cat breeding, sperm analysis for diagnostic purposes and sperm collection for cryopreservation and artificial fertilization, should be programmed when germ cell proliferation is more active and apoptosis is reduced.

## Data Availability

The data presented in this study are available in the Supplementary Materials Excel S1.

## Supplementary Materials

The following supporting information can be downloaded at: https://www.mdpi.com/article/10.3390/vetsci9080447/s1, Excel S1: PCNA and Apoptosis Data.
